# Efficacy and Safety of G2013 as a Novel Immunosuppressive Agent on Differentiation, Maturation and Function of Human Dendritic Cells

**Published:** 2017-02

**Authors:** Nazanin ARJOMAND FARD, Nakisa TABRIZIAN, Reza MIRZAEI, Jamshid HADJATI, Farzaneh TOFIGHI ZAVAREH, Ali Reza SALEHI NODEH, Abbas MIRSHAFIEY

**Affiliations:** 1. Dept. of Cellular and Molecular Biology, Kish International Campus, University of Tehran, Tehran, Iran; 2. Dept. of Immunology, School of Medicine, Tehran University of Medical Sciences, Tehran, Iran; 3. Dept. of Immunology, School of Public Health, Tehran University of Medical Sciences, Tehran, Iran

**Keywords:** NSAIDs, Anti-inflammatory agents, Dendritic cell, Immunosuppressive

## Abstract

**Background:**

The expanse of dendritic cells (DC) differentiation plays an important role in determining immune response. DC-based immunosuppressive drugs have notable side effects in increasing the risk of infectious diseases and cancers. G2013, as a novel anti-inflammatory and immunosuppressive agent, has been tested in experimental model of multiple sclerosis. The aim of this study was to conduct the safety property of G2013 on dendritic cells biology.

**Methods:**

The effect of G2013 on differentiation, maturation, and function of dendritic cells was examined at Tehran University in 2014. To investigate how G2013 affects human dendritic cells (DC) in a defined inflammatory environment, human peripheral blood mononuclear cells (PBMC) were isolated from healthy blood. Monocytes were then purified using anti-CD14 microbeads. Monocytes were treated with G2013 in two different doses (6 and 12 μg/well) along with adding granulocyte-macrophage colony-stimulating factor (GM-CSF) and interleukin-4 for inducing monocytes to immature DC and adding lipopolysaccharide for running DC maturation. Differentiation, maturation, and function of dendritic cells were examined with flow cytometry and ELISA.

**Results:**

G2013 therapy had no significant effect on CD83, CD86 and DR expression, as well as IL-10 and IL-12 cytokine levels and it, has no remarkable side on differentiation, maturation and function of dendritic cells in immature DC and mature DC process in vitro.

**Conclusion:**

G2013 is a safe agent with no adverse effect on differentiation, maturation, and function of dendritic cells. It may be recommended as a novel immunosuppressive agent with no or little side effect in increasing the risk of infectious diseases and cancers.

## Introduction

Dendritic cells (DC) are highly specialized and professional antigen-presenting cells (APC) that orchestrate the immune response via integration of a variety of incoming signals (1). They can be found in an immature state in the blood. Immature DCs can uptake antigens and migrate to lymph nodes where they interact with T cells and B cells. Then, in the presence of endogenous or exogenous inflammatory signals, DCs undergo maturation and initiate the adaptive immune response (2). DC maturation in response to TLR ligands and other pattern recognition receptors such as CD14 receptor is marked by induction of CD83, CD86, and MHCII (3). In other words, maturation of DCs is related to enhanced expression of co-stimulatory molecules such as CD80 and CD86, and MHC molecules (4). In addition, to expressing high levels of costimulatory molecules, mature DCs release large amounts of cytokines including IL-12, which can stimulate the Th1 immune response and IL-10 production. The release of IL-10 following up-regulation of costimulatory molecules and production of IL-12, blocks the DC maturation process, subsequently limiting the ability of DCs to initiate a Th1 response (5, 6). DC-based immune-suppressive drugs can suppress the progression of autoimmune diseases, however, their notable side effects in increasing the risk of cancer and infectious diseases should be considered (7, 8).

G2013 molecule is a novel designed drug with immunosuppressive property, classified as an non-steroidal anti-inflammatory drug (NSAIDs). It is a small molecule with low molecular weight extracted from alginate chemical hydrolysis method tested in experimental model of multiple sclerosis (MS). NSAIDs play an important role in the management of inflammatory diseases (9). During recent years, researchers have tried to identify safer and more effective types of anti-inflammatory and immunosuppressive drugs.

Since this drug had a potent immunosuppressive effect on MS model (10), we decided to test the efficacy and safety of G2013 as an immunosuppressive agent on differentiation, maturation and function of dendritic cells.

## Materials and Methods

G2013 was extracted from alginate powder (10).

### DC Differentiation and Maturation

In vitro human monocyte differentiation into DCs was based on the modified method. This experience was performed at Tehran University in 2014 (11). Six human peripheral blood mononuclear cells (PBMC) were isolated by Ficoll-Hypaque (Mediatech Cellgro) density gradient centrifugation from buffy coats provided from six human healthy blood donors. It was made by the informed consent protocol of the Declaration from Iranian Blood Transfusion Organization. Monocytes were purified from peripheral blood mononuclear cells using anti-CD14 microbeads (Miltenyi Biotec). The purified monocytes (>95% purity) were cultured at 37 °C in 24-well plates (700000 cells per well) in 3 mL of serum-free AIM V medium (Invitrogen) containing 100 ng/mL human granulocyte-macrophage colony-stimulating factor (GM-CSF) (peprotech) and human interleukin-4 (IL-4, 20 ng/mL, R&D Systems). Cells were cultured with two different doses of G2013 solution 6 μg/well as the low dose and 12 μg/well as the high dose and 6 wells were selected as control (non-treated by G2013). 0.5 mL of fresh medium with GM-CSF and IL-4 was added to the cell cultures on day 3. To induce DC maturation, lipopolysaccharide (LPS, 1 μg/mL, catalog no. L2654, Sigma-Aldrich) was added to cell cultures for 24 h on day 5, and again two doses of G2013 solution were added to the wells 4 h before adding the LPS.

### Detection of maturation and differentiation markers on cells

Harvested cells were washed twice with PBS and resuspended in 100 *μ*L PBS supplemented with 0.5% bovine albumin serum (BSA) and 0.1% sodium azide for staining. Cells were incubated for 10 min at room temperature with a FC receptor blocking solution (Bio Legend, San Diego, USA). Cells were then incubated at 4 °C for 30 min with the following monoclonal mouse anti-human antibodies (mAb): FITC-conjugated mAbs against cell surface molecules including CD83, CD14 and PE-conjugated mAbs against CD1a, CD86 and PE-CY5-conjugated mAbs against MHCII (eBiosciences, USA). In all experiments, isotype controls were included using an appropriate mAb of the same Ig class or subclass. After staining, cells were washed twice in PBS supplemented with 0.5% BSA and 0.1% sodium azide and resuspended in PBS with 0.99% paraformaldehyde. Flow cytometry was performed using a Cytomics FC-500 flow cytometer (Beckman Coulter), and all subsequent analyses were made with FlowJo software (Tree Star).

### Cytokine Measurement

DC cytokines production was detected respectively in supernatants of DCs culture (IL-12p70 and IL-10). Supernatants were collected and kept frozen at −70°C until use. Cytokine concentration was measured by enzyme-linked immunosorbent assay (ELISA) Kit (BenderMed system, Austria) according to the manufacturer’s instructions.

### Statistical Analysis

The results are expressed as means ± SD. All data were analyzed using the SPSS 20 application (Chicago, IL, USA). For non-normally distributed data, the nonparametric Friedman test was used for evaluating the statistical differences between three or four groups related to each other. Furthermore, the nonparametric Wilcoxon signed rank test was used to evaluate the differences between two groups with each other. A *P-*value<0.05 was considered significant. All calculations were performed using GraphPad Prism software 5 (GraphPad Software. Inc).

## Results

G2013 molecule is an immunosuppressive agent tested in experimental model of multiple sclerosis cells. To extend this study, we tested whether this drug has any effect on differentiation, maturation and function of dendritic cells in vitro or not.

### Differentiation

**Fig. 1:**
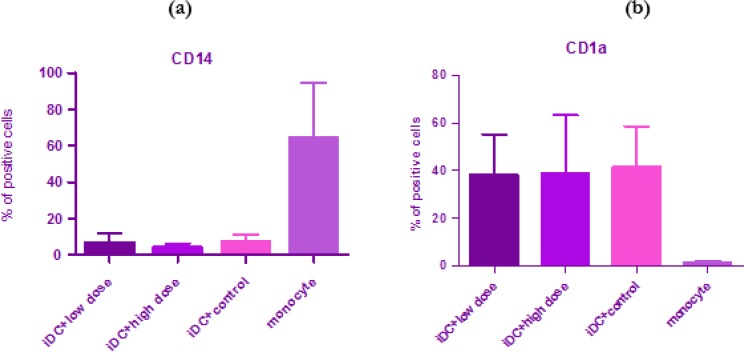
**(a, b):** Effect of G2013 with two doses, low (6 μg/well) and high (12 μg/well) on expression of CD14 and CD1a in monocyte and immature dendritic cell (IDC). There was no significant difference between control group and treated groups (*P*>0.05 was considered as nonsignificant)

To examine the expression level of CD14 and CD1a in monocyte and immature dendritic cells two different doses (6 and 12 μg/well) of G2013 were examined. There was no significant difference between control group and the groups treated with G2013 solution 6 μg/well and 12 μg /well in immature dendritic cells (Fig. 1).

### Maturation

As shown in Table 1, co-stimulatory molecules and MHC-II expressions were not significantly different between control group and immature dendritic cells treated with low and high doses of G2013 (6 μg/well and 12 μg/well).

**Table 1:** Effect of G2013 with two doses, low (6 μg/well) and high (12 μg/well)

**Variable**	**imDC Control**	**imDC Low dose**	**imDC High dose**	**mDC Control**	**mDC Low dose**	**mDC High dose**
CD83	6.56% ± 5.20	8.01% ± 6.05	10.1% ± 6.50	40.80% ± 38.11	46.60% ± 36.42	35.26% ± 38.18
CD86	25.66%± 7.53	28.02% ± 11.35	21.00% ± 12.00	74.76% ± 19.13	67.80% ± 14.32	72.23% ± 26.42
MHCII	69.53% ± 14.87	75.05% ± 15.10	70.12% ± 10.02	90.43% ± 12.12	88.50% ± 10.23	76.33% ± 2.37

### Function of DCs

**Fig. 2:**
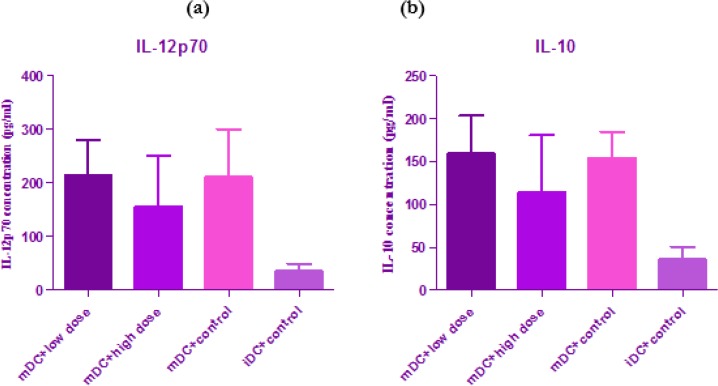
**(a, b):** Effect of G2013 with two doses, low (6 μg/well) and high (12 μg/well) on expression of IL-10 and IL-12p70 in mature dendritic cell. There was no significant difference between control group and treated groups (*P*>0.05)

Supernatants of cultured DCs were collected and ELISA measured the concentration of cytokines. There was no significant difference between control group and the groups treated by G2013 with 6μg/well (*P*>0.05) and 12 μg/well (*P*>0.05) in mature dendritic cells (Fig. 2).

## Discussion

G2013 molecule has been tested as an anti-inflammatory and a novel immunosuppressive agent in experimental model of multiple sclerosis. The molecular mechanism of therapeutic efficacy of this novel drug is based on its inhibitory effects on immune cells infiltration in inflammatory foci and significant reduction of the level of nitric oxide (NO) production in G2013-treated mice (10). During the past few years, the role of antigen presenting cells (APC) has been shown as a central axis for adaptive immune responses against extracellular bacteria, fungi, and other pathogens. DCs as an APC play a primary role in host defense and they are the only APCs capable of activating naive lymphocytes, which lead to initiate the protective immune responses against intracellular pathogens (12, 13). The degree of DC differentiation plays an important role in determining immune response (14). In humans, CD83, as immunoglobulin superfamily member, has been shown to be upregulated after DC maturation (15). In addition, the CD86 and MHCII levels usually increase significantly during DC maturation in humans and mice. In general, the expression of co-stimulatory molecules, CD80 and CD86, on DCs are known as a crucial secondary signal for the generation of effector T cells. Therefore, the up-regulation of CD80 and CD86 leads to enhance the activity of DCs. Lipopolysaccharide (LPS) increases CD80 and CD86 expression on DCs at inflamed sites (16, 17). CD80 and CD86 play a crucial role in the initiation and maintenance of an immune response. In the other word, the levels of all surface antigens mentioned above usually increase significantly during DC maturation (18). Moreover, Th1 and Th2 CD4^+^ T cell polarization are controlled by DC factors, such as the cytokines produced by them (19). Changes in the inflammatory environment can alter DC functions at all steps of their differentiation/maturation and effector functions (20).

This investigation showed that G2013 therapy had no significant effect on CD83, CD86, and DR expression as well as IL-10 and IL-12 cytokine levels. This result indicates G2013 is safe and well tolerated for treatment of various forms of autoimmune diseases. One of the most important therapeutic targets of researchers is DC suppression in the control of autoimmune diseases; however, the notable and dangerous side effects in progression and development of cancer and infectious diseases should be considered. In the present study, we have demonstrated the safety of G2013 molecule on differentiation, maturation, and function of dendritic cells.

## Conclusion

G2013 is a safe drug without any adverse effect on DC differentiation, maturation and function process in human immature and mature dendritic cells. Altogether, this drug has no or little side effect in increasing the risk of infectious diseases and cancers. Moreover, the assessment of adhesion molecules expression on the surface of immune cells following G2013 therapy could be recommended.

## Ethical considerations

Ethical issues (Including plagiarism, informed consent, misconduct, data fabrication and/or falsification, double publication and/or submission, redundancy, etc.) have been completely observed by the authors.
